# Carbon-Modified Attapulgite Composite for Rapid Rhodamine B Degradation: High Adsorption Capacity and Photo-Fenton Efficiency

**DOI:** 10.3390/ma19030554

**Published:** 2026-01-30

**Authors:** Naveed Karim, Tin Kyawoo, Saeed Ahmed, Weiliang Tian, Huiyu Li, Yongjun Feng

**Affiliations:** 1 State Key Laboratory of Chemical Resource Engineering, Beijing University of Chemical Technology, Beijing 100029, China; karim@buct.edu.cn (N.K.); huiyuli@mail.buct.edu.cn (H.L.); 2 Department of Biological & Chemical Sciences, University of Rasul, Mandi Bahauddin 50370, Pakistan; 3 College of Chemistry and Chemical Engineering, Tarim University, Alar 843300, China

**Keywords:** carbon-modified attapulgite, hydrothermal synthesis, photo-Fenton oxidation, wastewater treatment

## Abstract

A carbon-modified attapulgite composite (C-AATP@CTAB) was synthesized via the hydrothermal method using citric acid as the carbon source and cetyltrimethylammonium bromide (CTAB) as a surface modifier for efficient rhodamine B (Rh-B) removal. Carbon modification elevated the composite’s specific surface area (212 m^2^/g) and negative surface charge (−38.21 mV), significantly enhancing dye adsorption capacity to 666.66 mg/g—nearly double that of unmodified ATP variants (360.4–386.8 mg/g). Kinetic studies confirmed pseudo-second-order adsorption kinetics, attributed to hydrogen bonding and van der Waals interactions between Rh-B and the composite. Under photo-Fenton conditions, C-AATP@CTAB achieved 99.8% Rh-B degradation within 20 min, demonstrating superior catalytic performance in heterogeneous Fenton/photo-Fenton systems. This work establishes a low-cost, high-efficiency adsorbent-catalyst hybrid derived from low-grade attapulgite, offering promising avenues for sustainable wastewater treatment.

## 1. Introduction

The remediation of organic pollutants from water resources is critically important for safeguarding public health and preserving ecological security, positioning it as a prominent focus of contemporary research [1]. Among these pollutants, synthetic dyes warrant particular attention due to their toxic, carcinogenic, and mutagenic properties. These traits not only irritate the skin, eyes, and respiratory system but also disrupt plant physiological processes and metabolic activities in environmental settings, creating imbalances that ultimately inhibit growth [2]. A representative example is rhodamine B (Rh-B; C_28_H_31_ClN_2_O_3_), a widely recognized cationic dye used as a pigment and additive in pharmaceuticals, textiles, and nutritional products. Notably, Rh-B exerts detrimental effects on aquatic ecosystems even at low concentrations [3]. Despite its mutagenic and toxic profiles having driven bans in numerous countries, unregulated use in the dyeing industry persists, remaining a pressing environmental concern [4,5].

Numerous methods and technologies have been developed for high-efficiency degradation of Rhodamine B (Rh-B) [6,7]. Comparative studies reveal that the Fenton and photo-Fenton processes exhibit versatile capabilities for generating free radicals for dye degradation, making them more effective than conventional adsorption methods for removing organic dyes [8,9]. Fundamentally, this reaction belongs to advanced oxidation processes (AOPs), which are driven by iron ions that efficiently catalyze the breakdown of organic contaminants into simpler molecular compounds [10,11]. Nonetheless, conventional Fenton oxidation faces persistent challenges, including iron sludge formation, low hydrogen peroxide utilization efficiency, and difficulties with catalyst recovery and reuse [12,13]. To mitigate these limitations, carbon-based iron containing composites as heterogeneous Fenton-like catalysts have emerged as a promising alternative, offering high pollutant removal efficiency and low energy consumption [14]. However, the complexity and high cost of their synthesis processes further complicate scale-up for industrial manufacturing. Consequently, there is an urgent need to develop cost-effective, environmentally benign photo-Fenton catalysts that maintain high, stable catalytic performance over extended operation [15].

More recently, attapulgite (ATP), a crystalline hydrated magnesium silicate with a unique layer-chain structure and large specific surface area, has gained considerable attention as an effective support material in advanced oxidation processes [16]. Notably, natural ATP contains intrinsic iron impurities (${\mathrm{Fe}}^{2+}$/${\mathrm{Fe}}^{3+}$) derived from its geological formation, which can participate in redox reactions. For example, ATP from Baiyin, Gansu (China) contains approximately 8 wt.% iron, as reported in our previous work [14]. These naturally occurring iron species can catalyze the decomposition of hydrogen peroxide (H_2_O_2_) to generate hydroxyl radicals (•OH) through Fenton or photo-Fenton-like pathways [15]. In classical Fenton chemistry, ${\mathrm{Fe}}^{2+}$ reacts with H_2_O_2_ to produce •OH, whereas in photo-Fenton systems, light irradiation accelerates the ${\mathrm{Fe}}^{3+}$ → ${\mathrm{Fe}}^{2+}$ regeneration, thereby enhancing radical formation and overall degradation efficiency [16,17]. Consequently, ATP functions not only as a high-surface-area support but also as a naturally iron-containing material capable of facilitating heterogeneous Fenton and photo-Fenton catalytic reactions [18,19]. Moreover, its inherent hierarchical pore-size distribution further enhances its exceptional adsorption capacity [20]. Furthermore, ATP can be combined with carbon to fabricate functional catalysts that enhance the degradation efficiency of diverse organic dyes [21], including methylene blue, crystal violet, Rhodamine D, and Rhodamine B [22,23]. However, a critical limitation of these composite systems persists: catalytic activity is primarily governed by the carbon component, thereby preventing the full utilization of the attapulgite matrix’s intrinsic functional potential [24,25]. Recently, a carbon-modified pizza-like attapulgite (denoted C-AATP@CTAB) has been developed for the degradation of Rhodamine B (Rh-B). The abundant oxygen-containing functional groups on the carbon surface—including hydroxyl (–OH), carboxyl (–COOH), and amino (–NH_2_) groups—further provide a flexible platform for surface modification or the fabrication of complex composite materials. Despite these advancements, research gaps persist on ATP-carbon composites for photo-Fenton degradation—along with limited mechanistic insights into their performance.

In this work, we used cetyltrimethylammonium bromide (CTAB), which improves dispersion and carbon coating, while ethylenediamine adds amine groups to enhance surface reactivity and catalytic performance. All reagents were handled safely and removed after synthesis. Their use was essential to achieve the desired structure and functionality, via a hydrothermal method to produce carbon-modified ATP composites (C-A-ATP) for Fenton-like and Photo-Fenton catalysts for rapid rhodamine B degradation. The related key parameters and possible mechanism were carefully investigated. This approach contributes to the rational development of high-performance, environmentally friendly materials and provides a practical pathway for sustainable remediation of pollutants.

## 2. Results and Discussion

### 2.1. Morphology

**Figure 1:**
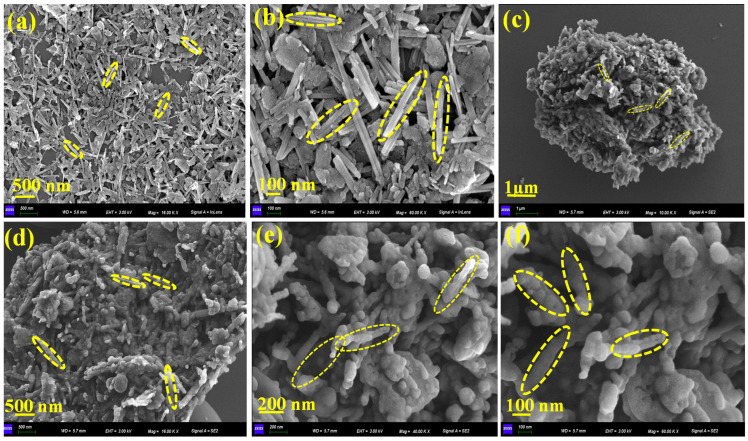
SEM images (**a**,**b**) A-ATP and (**c**–**f**) C-A-ATP@CTAB composites.

The morphological evolution of activated attapulgite (A-ATP) following surface modification was systematically investigated via scanning electron microscopy (SEM), with results shown in Figure 1a,b. Initially, pristine A-ATP exhibited a typical rod-like structure with smooth surfaces, loosely aggregated into bundles; individual rods measured approximately 30–50 nm in length. After composite formation (Figure 1c–f), the C-A-ATP@CTAB sample displayed significant morphological alterations: enhanced aggregation, increased surface roughness, and shortened rod-like structures embedded within a denser carbonaceous matrix. Notably, a distinctive “pizza-like” morphology emerged, which we attribute to the hydrothermal co-precipitation of citric acid-derived carbon with ATP in the presence of cetyltrimethylammonium bromide (CTAB). This process collectively promoted surface coverage, interfacial interaction, and partial encapsulation of ATP rods, resulting in altered texture and an apparent reduction in size. The observed decrease in visible rod length is not attributable to structural erosion or fragmentation—no direct evidence supports such a mechanism. Instead, we hypothesize that it stems from partial embedding of ATP rods within the carbonaceous matrix and from aggregation effects, which obscure their full dimensions in SEM images. To further corroborate composite formation and interfacial interactions, we employed X-ray photoelectron spectroscopy (XPS) and Fourier-transform infrared spectroscopy (FTIR). XPS analysis confirmed the presence of oxygen-containing functional groups (–OH, C=O, and C–O), indicating successful carbon incorporation into the ATP surface. Notably, the O 1s spectra exhibited shifts associated with –OH groups originating from both ATP and carbon, providing evidence for robust interfacial bonding.

### 2.2. Crystal Structure

**Figure 2:**
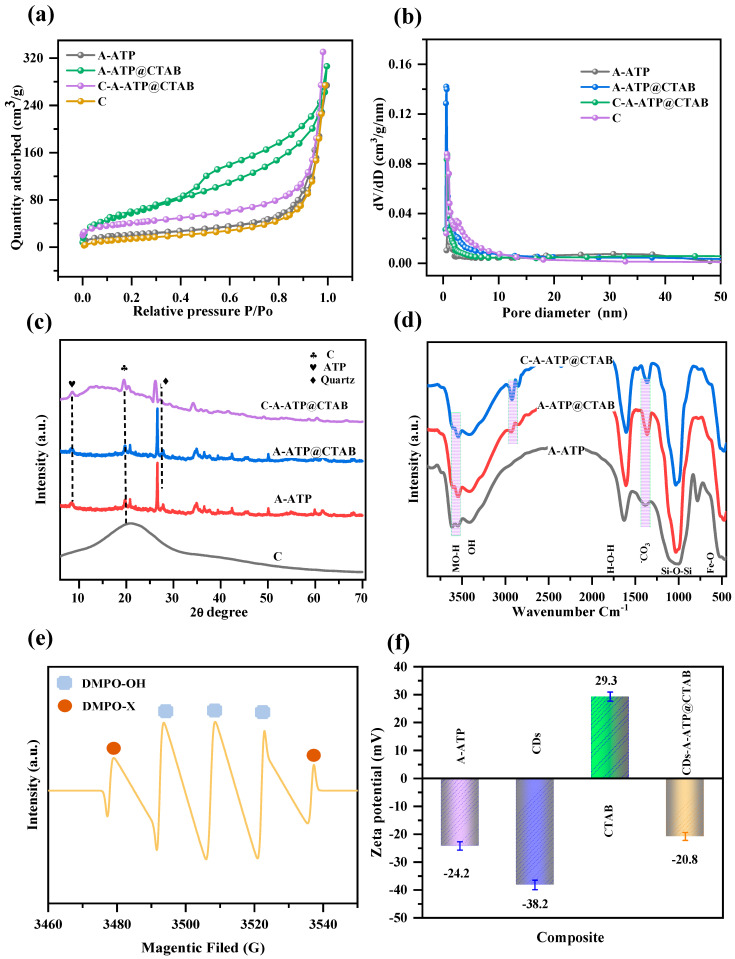
(**a**) N_2_ adsorption–desorption isotherms, (**b**) Pore diameter distribution curves (**c**) XRD pattern, (**d**) FTIR spectra, (**e**) radical test (EPR), and (**f**) Zeta potential charge analysis of a series of A-ATP, A-ATP@CTAB, and C-A-ATP@CTAB composites.

Figure 2c displays distinct diffraction peaks at specific 2θ angles in the XRD pattern of the C-ATP@CTAB nanocomposite. These peaks—located at 8.4°, 13.8°, and 19.8°—are assigned to the (110), (−110), and (040) crystallographic planes of attapulgite (A-ATP), respectively, confirming the preservation of the composite’s ordered crystalline structure. Additionally, a broad peak around 22.5°—consistent with previous studies—is attributed to amorphous carbon, verifying the successful integration of carbon into the composite matrix [25,26]. The absence of new diffraction peaks upon integrating A-ATP into the carbon network directly indicates the successful encapsulation of ATP within the carbon matrix [27]. Notably, while the characteristic peaks of bare ATP remain intact in C-ATP@CTAB, the intensity of the carbon peak is significantly diminished. This observation not only reinforces evidence for effective ATP encapsulation but also suggests that ATP integration subtly modulates the composite’s crystalline environment. Collectively, the retention of characteristic peaks from both ATP and carbon confirms the successful formation of the C-A-ATP@CTAB composite, with both components stably associated—carbon adhering to the ATP surface [28]. During carbonization, citric acid acted as a protective and stabilizing agent: it minimized ATP deformation during stirring and prevented structural collapse caused by ethanol evaporation during the drying step.

### 2.3. FT-IR Analysis

Figure 2e presents the FTIR spectra of both pristine A-ATP and the C-A-ATP@CTAB composite, enabling systematic identification of their key functional groups. A prominent peak at 3694 cm^−1^ is assigned to the O–H stretching vibrations of hydroxyl groups present in both A-ATP and the carbon component. A sharp peak at 3616 cm^−1^—attributed to the asymmetric stretching of Al–OH–Al in the aluminosilicate sheets of montmorillonite—was corroborated by previous studies [29]. Additional peaks corresponding to in-plane and out-of-plane bending vibrations of carbonate ions further indicate the successful incorporation of carbon and ATP clay into the nanocomposite [28]. A distinct peak at 1039 cm^−1^ arises from the stretching vibration of Si–O–Si bonds in the tetrahedral sheets of montmorillonite. Absorption bands at 2908 cm^−1^ and 2963 cm^−1^ are linked to C–H stretching vibrations of –CH_3_ and –CH_2_– groups, respectively. The peak at 1654 cm^−1^ originates from the bending vibration of coordinated or adsorbed water on the composite [29]. Peaks at 1031 cm^−1^ and 652 cm^−1^ are attributed to the stretching vibrations of Si–O bonds and the inverted tetrahedral SiO_4_ skeleton, respectively. Finally, peaks at 530 cm^−1^ and 580 cm^−1^ correspond to Fe–O stretching and bending vibrations, respectively. Collectively, these spectral assignments confirm the successful synthesis of the C-A-ATP@CTAB nanocomposite via the hydrothermal method [30,31].

### 2.4. Pore Structure Analysis

N_2_ adsorption–desorption isotherms were employed to characterize the samples further and evaluate the impact of carbon modification on the pore structure. Pristine ATP exhibited a Type-IV isotherm with H3-type hysteresis, consistent with the characteristic slit-like pores of traditional attapulgite [32]. Similarly, the C-A-ATP@CTAB composite displayed Type-IV isotherms with capillary condensation-induced hysteresis loops (Figure 2a,b), indicating preserved mesoporous structure analogous to pristine ATP. As summarized in Table 1, the BET-specific surface area (S_aβet_) increased sequentially for A-ATP (156 m^2^/g), A-ATP@CTAB (156 m^2^/g), and C-A-ATP@CTAB (212 m^2^/g)—with the latter reaching the highest value. Notably, the microporous surface area (S_mi_*c*_ro_) of C-A-ATP@CTAB contributed dominantly to the total S_aβet_ (212 m^2^/g). This enhancement in S_aβet_ is primarily attributed to the citric acid-derived carbon coating on ATP rods during hydrothermal carbonization, *c.f.*, Figure 2b. Specifically, the hydrothermally synthesized carbon modified the ATP surface, increasing surface roughness and introducing additional adsorption sites. Additionally, carbonization may penetrate ATP’s mesopores, further expanding the available adsorption volume.

Furthermore, these structural modifications likely induced partial narrowing or blockage of micropores in C-A-ATP@CTAB. Nevertheless, the composite retained a dominant microporous contribution to the total specific surface area—an outcome attributed to the synergistic effect of the carbon coating on ATP surfaces, which introduced abundant new adsorption sites to offset minor changes in pore access. In one word, our results conclusively verify the successful modification of carbon species onto A-ATP. Crucially, this deposition strategy enhanced surface roughness and elevated catalytic activity without compromising ATP’s inherent chemical composition or crystalline structure.

### 2.5. Zeta Potential

Zeta potential measurements (Figure 2f) revealed that all tested samples exhibited negative zeta potentials, consistent with predominantly negatively charged surfaces. In stark contrast, pure cetyltrimethylammonium bromide (CTAB) displayed a positive zeta potential (+29.3 mV), attributable to its quaternary ammonium groups [33]. Pristine attapulgite (A-ATP; −24.2 mV) and the citric acid-derived carbon component (−38.2 mV) inherently carried negative charges, a feature driven by their abundant carboxyl (–COOH) and hydroxyl (–OH) functional groups—these groups enable electrostatic repulsion in aqueous systems, a critical factor for colloidal stability [34]. For the composite series—from A-ATP to C-A-ATP@CTAB—zeta potentials ranged from −38.2 mV (A-ATP) to a maximum of +29.3 mV (approaching CTAB’s value). Notably, despite CTAB adsorption modifying surface chemistry, the final composite retained a net negative charge. This outcome underscores that the combined negative charge contributions from ATP and carbon dominate over CTAB’s positive charge, preserving long-range electrostatic repulsion. Such stability is essential for maintaining adsorption and catalytic degradation efficiency in wastewater treatment, as it prevents particle aggregation and maintains active-site accessibility.

### 2.6. EPR Analysis

To elucidate the degradation mechanism of Rhodamine B (RhB), we employed Electron Paramagnetic Resonance (EPR) spectroscopy to detect reactive oxygen species (ROS)—particularly hydroxyl radicals (•OH)—which are critical to the Fenton-like oxidation process [35]. A spin-trapping agent, 5,5-dimethyl-1-pyrroline N-oxide (DMPO), was used to capture and stabilize these highly reactive radicals, forming DMPO-OH adducts. The formation of these adducts and their involvement in RhB degradation were subsequently verified [36,37]. The characteristic EPR signal (1:2:2:1 intensity ratio) in Figure 2e provides conclusive evidence for •OH generation within the C-A-ATP@CTAB/H_2_O_2_ system. This strongly suggests that •OH radicals act as the primary oxidants, initiating and sustaining RhB degradation. Additionally, the sustained radical signal throughout the reaction indicates continuous •OH regeneration, likely attributed to efficient ${\mathrm{Fe}}^{2+}$/${\mathrm{Fe}}^{3+}$ redox cycling within the composite. This cycling not only enhances catalytic activity but also extends degradation efficiency—confirming that C-A-ATP@CTAB functions as a highly active and stable catalyst for advanced oxidation processes (AOPs) in wastewater treatment.

### 2.7. Thermal Analysis

**Figure 3:**
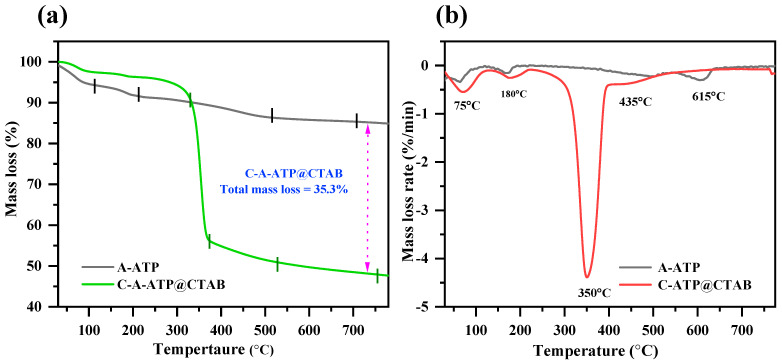
(**a**) Thermogravimetric analysis and (**b**) DTG outcomes of A-ATP, and C-A-ATP@CTAB, composite.

The thermal decomposition behavior of pristine attapulgite (A-ATP) and carbon-modified C-A-ATP@CTAB was investigated via thermogravimetric analysis (TGA) and derivative thermogravimetric analysis (DTGA) under an air atmosphere, with results presented in Figure 3a,b. Pristine A-ATP exhibited three distinct weight loss stages: a first stage (0–75 °C) corresponding to the evaporation of physically adsorbed water; a second stage (75–180 °C) attributed to the desorption of structurally bound water; and a third stage (180–615 °C) associated with the decomposition of hydroxyl groups and organic impurities inherent to ATP’s aluminosilicate structure. In contrast, the carbon-modified composite (C-A-ATP@CTAB) exhibited an additional fourth weight-loss stage, directly confirming the successful incorporation of carbon. The first two stages (0–75 °C and 75–180 °C) aligned with those of unmodified A-ATP, while the third stage (180–350 °C) arose from the decomposition of residual organic functional groups (e.g., leftover surfactants or citric acid derivatives from synthesis). The fourth stage (350–435 °C) involved the breakdown of carbonaceous networks and the volatilization of decomposition byproducts. Notably, carbon in the composite decomposed within 250–600 °C—a range attributed to deacetylation, volatilization, and removal of volatile byproducts from the carbon matrix. The total mass loss of C-modified A-ATP (35.3%) was significantly higher than that of pristine ATP (15.16%), a discrepancy directly explained by the added carbon content. This quantitative difference corroborates with SEM observations, which revealed ATP rods interconnected by a carbon network—further validating that carbon was successfully integrated into the composite structure [38,39].

### 2.8. XPS Analysis

**Figure 4:**
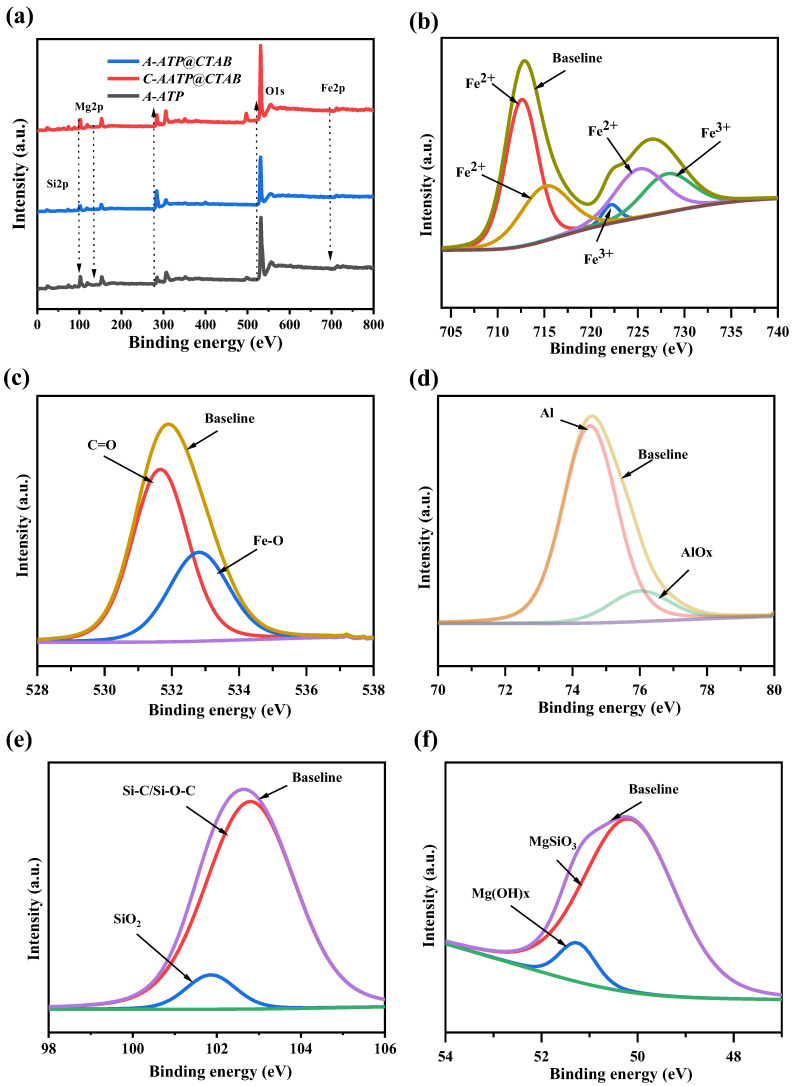
XPS Spectra of (**a**) A-ATP, A-ATP@CTAB, and C-A-ATP@CTAB nanocomposite; (**b**) Iron species; (**c**) Oxygen functionalities; (**d**) Aluminum species; (**e**) Magnesium functionalities; (**f**) Silicon species, and of C-A-ATP@CTAB composite.

X-ray photoelectron spectroscopy (XPS) was employed to investigate changes in the chemical environment of A-ATP following carbon incorporation [40]. Full-spectrum scans (Figure 4a) revealed consistent elemental compositions across all samples, including oxygen (O 1s), carbon (C 1s), silicon (Si 2p), magnesium (Mg 1s), and aluminum (Al 2p)—consistent with the preservation of core mineralogical elements. Carbon and oxygen were prioritized for detailed analysis, as their bonding states offer critical insights into the interfacial interactions between ATP and the carbon component. Deconvolution of the C 1s spectrum yielded three distinct peaks, attributed to C=C/C–C (284.9 eV, graphitic/aromatic carbon), C–O (286.4 eV, hydroxyl or ether linkages), and C=O (288.7 eV, carbonyl groups), respectively [41]. Notably, no significant shifts in peak binding energies were observed between pristine A-ATP@CTAB and C-A-ATP@CTAB—indicating that the chemical states of carbon (e.g., graphitic, hydroxyl, or carbonyl groups) remained largely unchanged after composite formation. Instead, the only measurable difference lay in the relative intensities of these C 1s peaks: the C-A-ATP@CTAB sample showed a marked increase in the proportion of C–O and C=O bonds compared to the initial A-ATP@CTAB, directly correlating with the incorporation of citric acid-derived carbon.

X-ray photoelectron spectroscopy (XPS) was used to quantify changes in carbon speciation (Figure 4) and to elucidate the chemical environment of key elements in C-A-ATP@CTAB. For carbon, the elevated C=O content (36.26%) in the composite—representing a 6.85% increase relative to pristine A-ATP—directly correlates with the surface –COOH groups of citric acid-derived carbon. This confirms the successful integration of the carbon component with A-ATP, as the carboxyl groups on citric acid remain intact after composite formation. Conversely, the reduction in C=C/C–C bonds (graphitic/aromatic carbon) is attributed to the partial disruption of A-ATP’s layered structure during the rigorous hydrothermal synthesis, which breaks down some of the inorganic carbonaceous domains in ATP.

Complementary analysis of surface composition and chemical states (supported by Table 2 and XPS survey spectra, Figure 4a) verified the presence of core elements (Al, Mg, Si, O, C, Fe) in both A-ATP and its composites. Deconvolution of the O 1s spectrum (Figure 4b) resolved three distinct oxygen species: carbonyl groups (C=O) at 531.2 eV, carbonate anions (${\mathrm{CO}}_{3}^{2-}$) at 532.4 eV, and metal-oxygen bonds in oxide phases (e.g., ATP’s aluminosilicate framework) at 534.9 eV. For iron, the Fe 2p spectrum (Figure 4c) identified ${\mathrm{Fe}}^{2+}$ (Fe 2p_3/2_ at 712.5 eV) and ${\mathrm{Fe}}^{3+}$ (Fe 2p_1/2_ at 724.9 eV), with further deconvolution revealing five sub-peaks: ${\mathrm{Fe}}^{2+}$ (712.5 eV, 715.1 eV) and ${\mathrm{Fe}}^{3+}$ (722.2 eV, 724.9 eV, 728.2 eV)—consistent with the mixed-valence state of iron in ATP and its composites. Aluminum (Al 2p, Figure 4d) showed peaks at 74.7 eV (elemental Al) and 75.8 eV (aluminum oxide, AlO_x_), while magnesium (Mg 2p, Figure 4f) and silicon (Si 2p, Figure 4e) displayed characteristic bonds for oxide/hydroxide phases (MgO, SiO_x_) and silicon-carbon interactions (Si–C/Si–O–C at 103.3 eV), respectively.

Critical to catalytic performance, the carbon binding energies (284.9 eV: C=O/carboxyl; 286.4 eV: C–O/C–N; 288.7 eV: graphitic C–C/C=C) confirmed the presence of both oxygenated (reactive for adsorption) and graphitic (stable support) carbon functionalities. The Fe 2p data further revealed a key catalytic mechanism: ${\mathrm{Fe}}^{2+}$ (81.08% before reaction) acts as the primary radical generator, producing hydroxyl radicals (•OH) for Rhodamine B (RhB) degradation via the Fenton reaction. Post-reaction, ${\mathrm{Fe}}^{2+}$ decreased to 75.23%, while ${\mathrm{Fe}}^{3+}$ increased—aligning with the expected oxidation of ${\mathrm{Fe}}^{2+}$ to ${\mathrm{Fe}}^{3+}$ during catalysis. Notably, ${\mathrm{Fe}}^{3+}$ persisted after five catalytic cycles, demonstrating that the composite’s active sites remain functional, and structural integrity is preserved.

Combined with Figure 4, Figure S2a–f and Table S1—which show retained reactive surface functionalities post-cycling—these findings underscore the material’s long-term stability and catalytic efficacy. The ${\mathrm{Fe}}^{2+}$/${\mathrm{Fe}}^{3+}$ redox cycling, coupled with preserved carbon-oxygen functionalities, ensures sustained adsorption and degradation of persistent organic pollutants (POPs) via an adsorption-Fenton oxidation pathway. This makes C-A-ATP@CTAB a promising candidate for environmental remediation.

### 2.9. Adsorption and Degradation for Rh-B

Initially, the performance of the synthesized C-A-ATP@CTAB nanocomposite in removing rhodamine B (Rh-B) from synthetic wastewater was evaluated to assess the impact of key operational parameters and to identify optimal treatment conditions. Subsequently, its efficacy was tested on real Rh-B-containing wastewater to validate practical applicability. Five critical parameters—contact time, nanocomposite dosage, initial Rh-B concentration, pH, and temperature—were systematically investigated to define ideal conditions for Rh-B removal.

**Figure 5:**
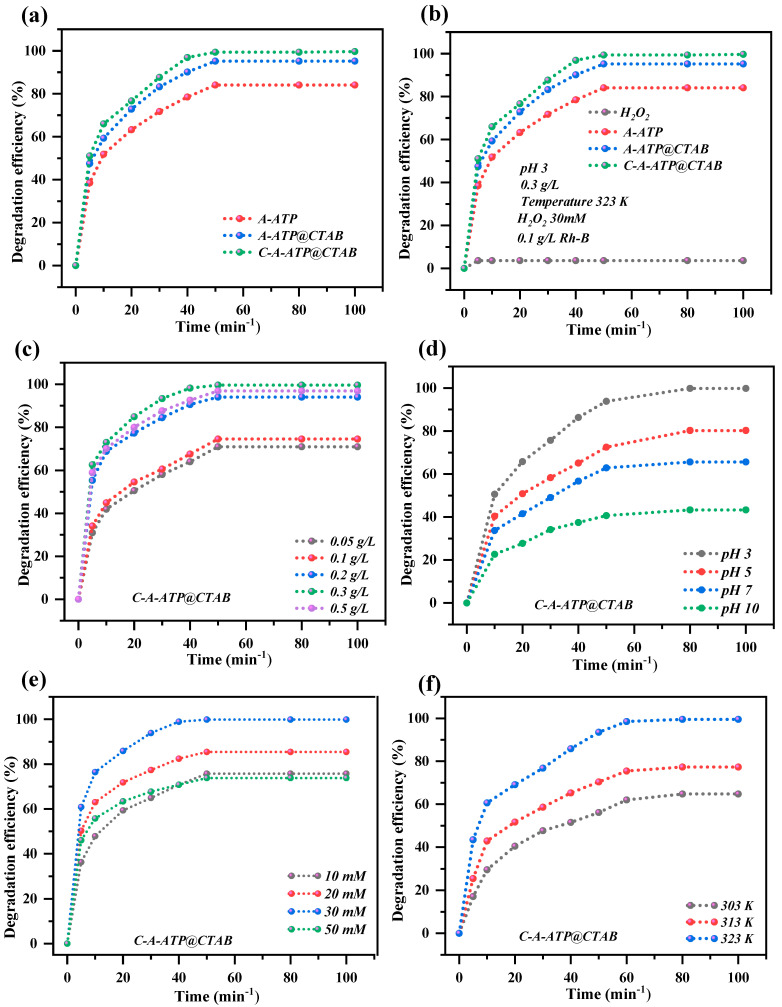
(**a**) Adsorption (removal efficiency) (**b**) degradation efficiencies of A-ATP, A-ATP@CTAB, C-A-ATP@CTAB, and C (**c**) dosage effect, (**d**) pH study, (**e**) H_2_O_2_ concentration, and (**f**) temperature effect. The experiment was conducted at a dosage of 0.3 g/L, pH of 3, temperature of 323 K, and H_2_O_2_ concentration of 55 mM for coupling adsorption and Fenton-like oxidation of Rh-B 0.1 g/L.

As shown in Figure 5a, Rh-B adsorption efficiency increased rapidly with extended contact time, achieving > 60% removal within the first 120 min and Figure 5b shows degradation efficiency of 96% in 50 min. No significant improvement was observed beyond this point, indicating that the composite’s adsorption capacity was saturated or residual Rh-B was negligible. This aligns with photo-Fenton (complete removal in 20 min) and Fenton-like oxidation (complete removal in 50 min) benchmarks. A 20 min contact time was thus selected for subsequent experiments.

Rh-B degradation increased with higher dosages, attributed to the greater number of adsorption sites available as the surface area rose. A sharp improvement in removal was seen between 0.5 and 0.05 g/L, with marginal gains at 0.5 g/L before plateauing—signaling equilibrium (Figure 5c). An optimal dosage of 0.3 g/L was determined for an initial Rh-B concentration of 0.1 g/L, balancing efficiency and material economy. The composite exhibited pH-independent performance across 3–10 and consistent degradation across tested temperatures (Figure 5d). The pH plays a critical role in regulating both the surface charge of the catalyst and the ionization state of Rhodamine B (Rh-B). Within the acidic to near-neutral range (pH 3–7), the composite surface remains predominantly positively charged, while Rh-B exists mainly in its neutral or zwitterionic form; consequently, electrostatic interactions are relatively weak. Under these conditions, adsorption and degradation are primarily governed by π–π interactions, hydrogen bonding, and van der Waals forces, resulting in high removal efficiency. In contrast, temperature exerts a more pronounced influence on the reaction kinetics. The apparent rate constant (*k_app_*) increased exponentially with increasing temperature, rising from 0.015 min^−1^ at 303 *k_app_* to 0.060 min^−1^ at 313 *k_app_* and further to 0.150 min^−1^ at 323 *k_app_* (Figure 5f, inset), which is consistent with Arrhenius-type behavior. After full optimization of the operational parameters, the highest Rh-B adsorption capacity reached 666.66 mg/g (Table 3). To elucidate reaction dynamics, the influence of temperature, H_2_O_2_ concentration, ATP dosage, initial Rh-B concentration, and pH on Fenton-like oxidation was evaluated (Figure 5c,d). The pseudo-second-order kinetic model provided an excellent linear fit (Figure 5d), confirming chemisorption as the rate-limiting step.

For Fenton experiments, the dye solution (initial Rh-B: 0.5 g/L) was adjusted to pH 3–10 using NaOH/CH_3_COOH, followed by adding 0.3 g/L C-A-ATP@CTAB. The mixture was equilibrated, then 55 mM H_2_O_2_ was added to initiate degradation (Figure 5).

### 2.10. Degradation of Rh-B

The adsorption and degradation performance of pristine attapulgite (A-ATP), CTAB-modified A-ATP (A-ATP@CTAB), carbon-modified C-A-ATP@CTAB, and standalone carbon for rhodamine B (Rh-B) was systematically evaluated under varying dosages, pH, and H_2_O_2_ concentrations (Figure 5a–f). Over 120 min (adsorption) and 100 min (degradation), their efficiencies were: A-ATP (36.4% adsorption/81.6% degradation), A-ATP@CTAB (41.6%/91.6%), C-A-ATP@CTAB (63.1%/99%), standalone carbon (53.4% adsorption only), and H_2_O_2_ alone (1.5% degradation). As summarized in Table 3, C-A-ATP@CTAB exhibited the highest combined adsorption/degradation efficiency and adsorption capacity, outperforming all counterparts. The dramatic improvement in C-A-ATP@CTAB (vs. A-ATP@CTAB: 41.6%/91.6% → 63.1%/99%) stems from synergistic integration: ATP provides a high-specific-surface-area adsorbent for initial Rh-B uptake, while citric acid-derived carbon introduces catalytically active sites for Fenton-like •OH generation. This dual mechanism enhances mass transfer and extends the degradation pathways—addressing the limitations of standalone materials. In contrast, standalone H_2_O_2_ (1.5% degradation) failed to generate sufficient radicals without a catalyst, and standalone carbon (53.4% adsorption) lacked the catalytic activity to drive efficient degradation, highlighting the necessity of the composite structure.

**Figure 6:**
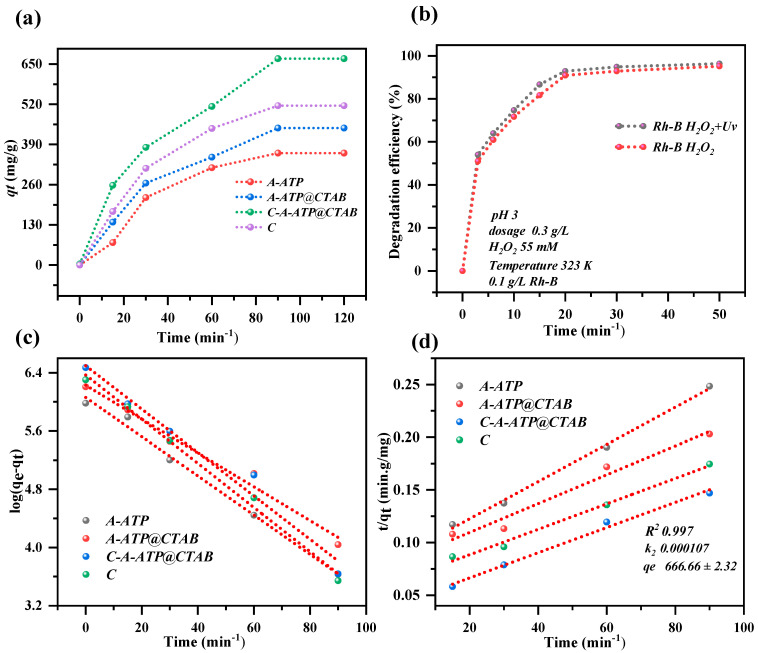
(**a**) Adsorption kinetic, (**b**) degradation efficiency (%), (**c**) pseudo-first-order, (**d**) pseudo-second-order for Rh-B. The experiment was conducted at a dosage of 0.3 g/L mg/L, pH of 3, temperature of 323 K, H_2_O_2_ volume of 250 mL and 0.1 g/L Rh-B.

Notably, adding C-A-ATP@CTAB to an H_2_O_2_-containing Rh-B solution boosted degradation to 99.6% within 20 min, demonstrating rapid and effective pollutant removal. Kinetic analysis (Figure 6a–d) confirmed the composite follows both first-order and pseudo-second-order models, with the pseudo-second-order rate constant indicating chemisorption as the rate-limiting step. This aligns with optimal reaction conditions, where active sites (ATP’s hydroxyl groups and carbon’s C=O/C–O bonds) are fully utilized.

These results underscore C-A-ATP@CTAB’s exceptional performance in H_2_O_2_-assisted Rh-B degradation, driven by its dual adsorption-catalysis mechanism and optimized active site availability. The composite’s ability to bridge adsorption (ATP) and catalysis (carbon) makes it a superior candidate for practical wastewater treatment.

### 2.11. Photo-Fenton Degradation

The adsorption and photo-Fenton degradation performance of C-A-ATP@CTAB for rhodamine B (Rh-B) were evaluated via batch experiments. In each run, a predetermined amount of the nanocomposite was added to a conical flask containing 100 mL of Rh-B solution (100 mg/L, mass concentration). H_2_O_2_ was then added, and the mixture was transferred to a closed photochemical reaction system (equipped with a magnetic stirrer, a 300 W xenon lamp, and a timer). Post-reaction, the nanocomposite was separated via centrifugation (9000 rpm, 5 min), and residual Rh-B in the filtrate was quantified by UV-Vis spectrophotometry.

Rh-B removal was systematically tested under varying dosages, pH levels, and temperatures. Initial experiments at pH 3 (100 mg/L Rh-B) revealed that C-A-ATP@CTAB significantly outperformed pristine A-ATP, A-ATP@CTAB, and standalone UV treatment after 30 min of dark adsorption. This superior degradation capacity stems from synergistic surface interactions: the A-ATP framework provides a high-specific-surface-area adsorbent for initial Rh-B uptake, while the incorporated citric acid-derived carbon introduces catalytically active sites for photo-Fenton •OH generation.

**Figure 7:**
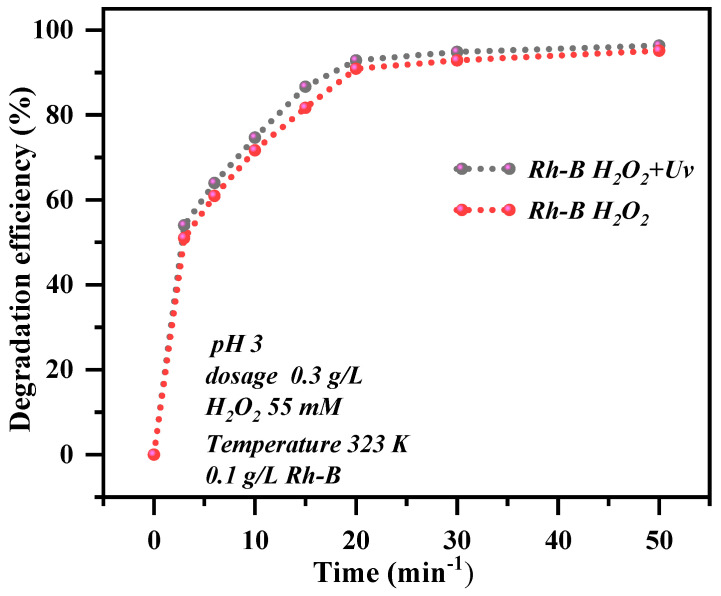
Comparison graph of Photo-Fenton and Fenton-like of C-A-ATP@CTAB for Rh-B (optimal study at dosage of 0.3 g/L, 323 K, pH 3, and 55 mM H_2_O_2_ and Rh-B 0.1 g/L).

As summarized in Figure 7, degradation efficiencies varied markedly: A-ATP@CTAB achieved 99.8% Rh-B removal in 20 min, yet C-A-ATP@CTAB exhibited an even higher photo-degradation efficiency. This substantial improvement underscores the critical role of carbon incorporation in enhancing composite performance. Notably, C-A-ATP@CTAB also showed a significantly faster photo-Fenton degradation rate than other materials—including in Fenton-like oxidation—confirming a synergistic effect between ATP’s adsorption capacity and carbon’s catalytic activity. These findings highlight C-A-ATP@CTAB’s exceptional potential for Rh-B removal. By bridging high-efficiency adsorption (ATP) with catalytic radical generation (carbon), the composite addresses the limitations of standalone materials and emerges as a promising candidate for environmental remediation.

### 2.12. Comparative Study of Heterogeneous and Photo-Fenton

It is well-documented that light irradiation accelerates the degradation rate of organic pollutants in Fenton oxidation processes [42,43,44,45]. Building on this, the present study systematically investigated the influence of visible and ultraviolet (UV) light on Fenton-based Rh-B degradation using the high-performance C-A-ATP@CTAB composite, selected for its exceptional catalytic activity. Experiments were conducted under two conditions: with H_2_O_2_ (to activate Fenton/photo-Fenton reactions) and without H_2_O_2_ (to isolate light effects). As illustrated in Figure 7 and quantified in Table S2, photo-Fenton (UV + H_2_O_2_ + C-A-ATP@CTAB) and Fenton-like (no UV + H_2_O_2_ + C-A-ATP@CTAB) processes were evaluated for Rh-B removal. In the presence of H_2_O_2_, photo-Fenton demonstrated significantly faster Rh-B degradation than Fenton-like oxidation. Notably, without H_2_O_2_, light irradiation alone only marginally accelerated Rh-B elimination, with degradation rates rising slowly over time—highlighting the critical role of H_2_O_2_ in generating reactive oxygen species (ROS) for pollutant breakdown. Quantitative comparisons revealed that under UV irradiation, Rh-B degradation reached 98.6% within 20 min (photo-Fenton), whereas Fenton-like oxidation (no UV) achieved only 85% degradation in the same timeframe (Table S2). This stark difference underscores that light synergies with H_2_O_2_ and the composite’s catalytic sites to enhance •OH generation, a key driver of rapid Rh-B mineralization. Collectively, these results confirm that photo-Fenton is the most favorable and optimal pathway for Rh-B degradation using C-A-ATP@CTAB, leveraging light to amplify the composite’s catalytic efficiency and accelerate pollutant removal.

### 2.13. Fe Leaching

Throughout the reaction, the catalyst inevitably releases iron species. Analysis of dissolved iron leaching revealed that ${\mathrm{Fe}}^{2+}$ concentrations during Rh-B degradation were higher than under other conditions—an expected outcome, as ${\mathrm{Fe}}^{2+}$ leaching is more pronounced in acidic environments, a factor that enhances Rh-B degradation rates in Fenton reactions [46]. Across all samples, ${\mathrm{Fe}}^{2+}$ levels increased over time, peaking at 60 min—coinciding with ~100% Rh-B removal. ICP data tracked this trend, showing a modest increase from 1.32 mg/L to 1.42 mg/L, indicating minimal overall iron leaching. Text S1 (Reusability) and Figure S2 further demonstrate the reusability of a catalysis, which indicate its stability during the degradation process.

Total iron ion concentrations also rose gradually, remaining higher than ${\mathrm{Fe}}^{2+}$ levels—consistent with the dominance of heterogeneous Fenton processes in Rh-B degradation [47]. Notably, total dissolved iron (${\mathrm{Fe}}^{2+}$ + other iron species) concentrations decreased with increasing carbon content in the C-A-ATP@CTAB nanocomposite, suggesting that carbon effectively mitigates iron leaching. Additionally, total iron leaching was enhanced by exposure to light or ultrasound, with leaching rates following the order: UV + Fenton > UV > Fenton. This indicates that elevated Fenton activity under light or photo-Fenton treatment correlates with increased iron ion release. Text S2 (Degradation scheme of Rh-B) and Figure S3 further illustrate the degradation mechanism via mass spectrometric analysis.

### 2.14. Langmuir-Hinshelwood Kinetics

**Figure 8:**
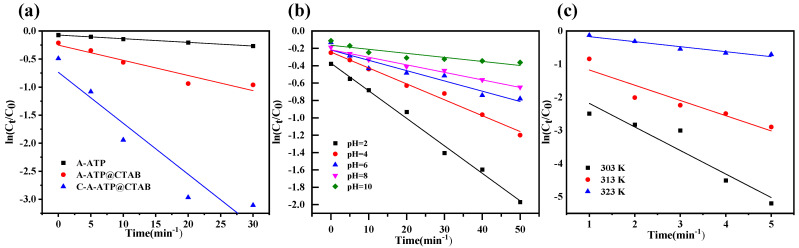
A change in: (**a**) materials amount, (**b**) temperature, (**c**) pH study for Langmuir–Hinshelwood kinetic degradation of Rh-B (optimal dosage 0.3 g/L, H_2_O_2_ 30 mM, temperature 323 K, pH 3, and Rh-B 0.1 g/L).

The photocatalytic degradation kinetics were described by the Langmuir–Hinshelwood model, ln (*C_t_*/*C*_0_) = *k_app_* where *C*_0_ and *C_t_* are the initial and time-dependent dye concentrations, respectively, and *k_app_* is the apparent first-order rate constant reflecting the surface-controlled photocatalytic reaction. The heterogeneous photocatalytic degradation kinetics at an initial dye concentration of 100 mg dm^−3^ were evaluated using the Langmuir–Hinshelwood model. Under these conditions, the linearized Langmuir–Hinshelwood equation was applied, and the apparent rate constant (*k_app_*) was obtained from the slope of the ln (*C_t_*/*C*_0_) versus degradation time plot. As summarized in Table 4 and Figure 8, all studied samples—A-ATP, A-ATP@CTAB, and C-A-ATP@CTAB—exhibited excellent linearity with high determination coefficients (*R*^2^ = 0.99), confirming that the photocatalytic degradation process follows the Langmuir–Hinshelwood mechanism [48,49]. The calculated *k_app_* values were 0.007 min^−1^ for A-ATP, 0.037 min^−1^ for A-ATP@CTAB, and 0.145 min^−1^ for C-A-ATP@CTAB, indicating a substantial enhancement in degradation efficiency after surface modification. Among the catalysts, C-A-ATP@CTAB showed the highest photocatalytic activity, which can be attributed to improved surface characteristics and more effective interaction between the catalyst surface and dye molecules, leading to faster surface oxidation reactions. These results demonstrate that, at a fixed initial concentration, the Langmuir–Hinshelwood model reliably describes the degradation kinetics and enables meaningful comparison of photocatalytic performance among the different catalysts (Table 5).

## 3. Materials and Methods

### 3.1. Chemicals

Chemicals were obtained from Shanghai Aladdin Bio-Chem Technology Co., Ltd. (China) and included citric acid (C_6_H_8_O_7_), hydrogen peroxide (H_2_O_2_, 30%), Rhodamine-B (C_28_H_31_ClN_2_O_3_), and cetyltrimethylammonium bromide (CTAB, C_19_H_42_BrN, 99.5%). All reagents were of analytical grade and were used without further purification. Attapulgite (ATP) was sourced from Baiyin City in Gansu Province, China. Deionized water used was made in the laboratory.

### 3.2. Synthesis of Carbon-Modified Attapulgite

First, 2 g of attapulgite (ATP) was dispersed in deionized water via ultrasonic treatment for 60 min to ensure thorough particle dispersion. Separately, cetyltrimethylammonium bromide (CTAB) was dissolved in deionized water at a ratio of 0.6 g CTAB per gram of ATP. This CTAB solution was gradually added to the ATP dispersion, followed by vigorous stirring for two hours to promote CTAB adsorption onto ATP surfaces. This modification functionalized ATP particles, enhancing their surface properties to facilitate subsequent composite formation.

Next, 4.2 g of citric acid was dissolved in 40 mL of deionized water, and 1.34 mL of ethylenediamine was added. The mixture was transferred to a 100 mL Teflon-lined stainless-steel autoclave, sealed, and heated at 200 °C for five hours. After cooling to room temperature, the carbon product was centrifuged and dried at 80 °C for 24 h.

**Figure 9:**
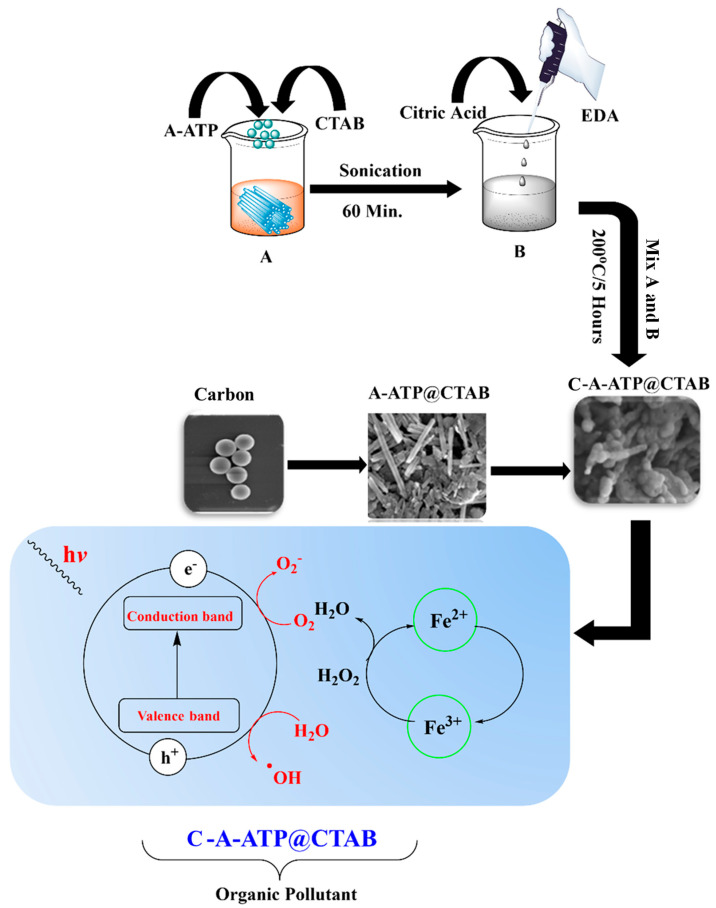
Fabrication of carbon-modified attapulgite coupling adsorption, and Photo-Fenton degradation for Rh-B.

Finally, 0.5 g of the prepared carbon was added to 1 g of the ATP-CTAB suspension, the mixture was hydrothermally treated at 180 °C for 12 h. During this process, carbon was uniformly distributed on ATP surfaces and encapsulated. The introduction of carbon enriched the ATP with oxygen-containing functional groups (–COOH, –OH), improving its hydrophilicity, surface charge, and active sites—critical for enhanced adsorption and catalytic performance. The resulting composite, designated C-AATP@CTAB, was thoroughly washed with deionized water, dried at 80 °C, and stored for further characterization and application studies. Figure 9 shows the schematic formation of the nanocomposite.

### 3.3. Instrumentation

X-ray Diffraction (XRD) Analysis: Powder XRD patterns were collected using a SHIMADZU XRD-6000 diffractometer equipped with a Cu Kα radiation source (λ = 1.5406 Å) operated at 40 kV. Data were acquired over a 2θ range of 5–70° to characterize crystalline phase composition. Fourier Transform Infrared (FT-IR) Spectroscopy: FT-IR spectra were recorded on a Bruker Vector 22 spectrometer via the potassium bromide (KBr) pellet method, with a sample-to-KBr mass ratio of 1:100. Spectra were collected in the mid-infrared region (4000–500 cm^−1^) to identify functional group assignments. Morphological Characterization: Surface morphology was analyzed using a Zeiss Supra 55 scanning electron microscope (SEM) and a JEOL JEM-2020 transmission electron microscope (TEM). SEM imaged surface features, while TEM resolved particle structure and dispersion. Nitrogen Adsorption–Desorption Analysis: Low-temperature (77 K) N_2_ adsorption–desorption isotherms were measured on a Micrometrics ASAP 2460 analyzer. Samples were degassed at 120 °C for 3 h to remove adsorbed moisture and contaminants prior to testing. Specific surface area (S_aβet_), pore volume, and pore size distribution were calculated from the adsorption isotherm using the Brunauer–Emmett–Teller (BET) method.

### 3.4. Batch Removal

Batch adsorption–desorption and degradation experiments for rhodamine B (Rh-B) were conducted in 250 mL conical flasks. Solutions were stirred in an orbital shaker with a temperature-controlled water bath (dark conditions) at 150 rpm to reach adsorption equilibrium. For degradation tests, the catalyst (C-A-ATP@CTAB, 0.05–0.5 g/L) was added to 100 mL of 100 mg/L Rh-B solutions at pH 3–12. The mixtures were equilibrated for 120 min to balance adsorption and desorption. Degradation was initiated by adding 55 mM H_2_O_2_ (from a 30% w/w stock solution) at 323 K (50 °C) to trigger the Fenton-like reaction. Rh-B concentrations were monitored over time at its maximum absorption wavelength (λ_max_ = 556 nm). Post-reaction, the spent composite was recovered via centrifugation (9000 rpm, 5 min). All experiments were performed in triplicate to ensure reproducibility. The spent C-A-ATP@CTAB was reused to evaluate regeneration potential. Subsequent degradation tests with the recovered sorbent followed the same optimization conditions (pH, catalyst dosage, H_2_O_2_ concentration).

For the photo-Fenton process, identical conditions were applied, but reactions were conducted under UV-visible irradiation (365 nm, 300 W UV lamp positioned above the mixture) to enhance efficiency. Continuous stirring ensured uniform mixing, and degradation was monitored periodically. The synergistic effect of light irradiation and hydroxyl radical (•OH) generation was hypothesized to accelerate Rh-B mineralization. Adsorption and degradation efficiencies were calculated using Equation (1), and degradation rates were determined via this equation [25]. Further studies with the regenerated sorbent used Equation (2) to compute degradation rates.

(1) $$R\;(\%)=\frac{C_{0\;}-C_{t\;}}{C_{0\;}}\;\times \;100$$

where *R* (%) is the removal efficiency of Rh-B and at time *t* (min), *C*_0_ (mg L^−1^) and *C_t_* (mg L^−1^) are the initial concentration and the concentration at time *t* (min) of Rh-B, respectively.

(2) $$q_{e\;}=\frac{\left(C_{0}-C_{e}\right)\times V}{m}$$

(3) $$ln\;(q_{e}-q_{t\;})=lnq_{e}-k_{1}t$$

(4) $$\frac{t}{q_{t\;}}=\frac{1}{k_{2}q_{e}^{2}}+\frac{t}{q_{e\;}}$$

where *q_e_* (mg/g) and *q_t_* (mg/g) represent the adsorption capacities of Rh-B at equilibrium and at time *t* (min), respectively. *C_t_* is the residual Rh-B concentration in the solution after *t* minutes of adsorption, while *C_e_* is the concentration of Rh-B at equilibrium. *k*_1_ (min^−1^) and *k*_2_ (mg (g·min)^−1^) denote the adsorption rate constants for the pseudo-first-order and pseudo-second-order kinetic models, respectively.

## 4. Conclusions

In conclusion, the hydrothermally synthesized C–A–ATP@CTAB composite, prepared using citric acid as a carbon precursor and CTAB as a surface-modifying agent, exhibited outstanding adsorption capability as well as highly efficient Fenton-like and photo-Fenton catalytic degradation of Rhodamine B (Rh-B). The material achieved a high adsorption capacity of 612 mg g^−1^, with the adsorption behavior well described by the pseudo-second-order kinetic model, indicating a chemisorption-dominated process governed by hydrogen bonding and van der Waals interactions. Under optimized Fenton conditions (catalyst dosage of 0.5 g L^−1^ and H_2_O_2_ concentration of 55 mM), the composite accomplished 99.8% Rh-B degradation within 20 min, while photo-Fenton irradiation further shortened the degradation time to 12 min. Although light irradiation provided only a moderate enhancement, this result highlights the strong intrinsic Fenton-like activity of the catalyst under dark conditions. The presence of naturally occurring iron species in attapulgite promotes efficient hydroxyl radical generation, while the synergistic interaction between the carbon phase and ATP enhances electron transfer and suppresses particle agglomeration. Overall, these findings demonstrate that the C–A–ATP@CTAB composite is a cost-effective and environmentally benign catalyst with high dye removal efficiency under mild reaction conditions, making it a promising candidate for practical wastewater treatment applications.

## Figures and Tables

**Table 1:** Pore structure parameters of A-ATP, A-ATP@CTAB, and C- A-ATP@CTAB nanocomposites.

Sample		Surface Area (m^2^/g)	Total Pore Volume (mL/g)	Average Pore Size (nm)
1	A-ATP	156	0.57	14.76
2	A-ATP@CTAB	156	0.16	6.05
3	C-A-ATP@CTAB	212	0.51	11.97
4	Carbon	221	0.35	7.61

**Table 2:** XPS Survey analysis, chemical composition (wt.%), of a series of A-ATP, A-ATP@CTAB, and A-C-A-ATP@CTAB composites.

Composite Materials	% Atomic of Elements					
	C	O	Al	Si	Mg	Fe
A-ATP	16.47	53.16	7.49	17.83	3.02	2.04
A-ATP@CTAB	22.39	49.90	6.15	15.28	3.70	2.59
C-ATP@CTAB	48.00	35.20	4.18	9.53	1.56	1.55

**Table 3:** Kinetic parameters of A-ATP and A-ATP@CTAB and carbon modified C-A-ATP@CTAB and carbon for coupling adsorption and Fenton-like oxidation of Rh-B.

Samples	*q_e_*_, exp_ (mg/g)	Pseudo-First Order			Pseudo-Second Order		
		*q_e_* (mg/g)	*k*_1_	*R* ^2^	*q_e_*	*k*_2_	*R* ^2^
A-ATP	362.32 ± 2.76	369.44 ± 1.89	−0.01667	0.992	416.66 ± 2.14	0.000128	0.915
A-ATP@CTAB	443.34 ± 2.35	463.58 ± 1.71	−0.00842	0.954	416.66 ± 1.92	0.00001	0.974
C-ATP@CTAB	612.08 ± 2.89	658.52 ± 1.84	−0.01294	0.974	666.66 ± 2.32	0.000107	0.997
Carbon	515.58 ± 2.65	528.47 ± 2.45	−0.14677	0.997	588.23 ± 2.75	0.00001	0.993

**Table 4:** Langmuir-Hinshelwood kinetic A-ATP composites for degradation of Rh-B.

Langmuir-Hinshelwood Kinetic		
Sample	*k_app_*	*R* ^2^
A-ATP	0.007	0.99
A-ATP@CTAB	0.037	0.99
C-A-ATP@CTAB	0.145	0.99

**Table 5:** Comparative study of the removal efficacy of organic dyes.

Catalyst	Dyes	Reaction Conditions		Degradation Efficiency (time)	*k* (min^−1^)	Mechanism	Ref.
		[Dyes] (g/L^−1^)	[Catalyst] (g/L^−1^)				
CuO–CeO_2_–Fe_2_O_3_/ATP	Methylene Blue	100	1.0	99.83% (60 min)	0.052 min^−1^	Heterogeneous Fenton systems	[50]
Fe-Cu/ATP-GO	Rhodamine-B	100	0.4	99.9% (50 min)	0.061 min^−1^	Photo-degradation	[51]
Modified APT	Methylene Blue	150	1.0	97.5% (120 min)	0.023 min^−1^	Adsorption-degradation	[52]
ATP-g-C_3_N_4_/SnFe_2_O_4_/Bi_2_WO_6_	Rhodamine-B	100	0.4	99.9% (60 min)	0.049 min^−1^	Photo-degradation	[32]
C-A-ATP@CTAB	Rhodamine-B	100	0.3	99.8% (50 min)	0.059 min^−1^	Adsorption and Fenton-like degradation	This work
C -A-ATP@CTAB	Rhodamine-B	100	0.3	99.8% (20 min)	0.145 min^−1^	Photo Fenton degradation	This work

## Data Availability

The original contributions presented in this study are included in the article/Supplementary Materials. Further inquiries can be directed to the corresponding author.

## Supplementary Materials

The following supporting information can be downloaded at: https://www.mdpi.com/article/10.3390/ma19030554/s1, Figure S1: After degradation (**a**) types of iron; (**b**) oxygen functionalities; (**c**) aluminum and AlO_x_; (**d**) nature of silicon functional groups; (**e**) nature of magnesium functionalities; (**f**) carbon functionalities and in C-A-ATP@CTAB composite; Figure S2: Reusability of CDs-A-ATP@CTAB nanocomposite after washing with ethanol and neutralization (**a**−**d**), XPS, XRD, FTIR, and reused cycle efficacy; Figure S3: LC-MS possible degradation pathways of Rh-B in the C-A-ATP@CTAB; Table S1: Binding energy peak percentage of ${\mathrm{Fe}}^{2+}$ and ${\mathrm{Fe}}^{3+}$ in A-ATP@C composite; Table S2: Comparison study for Heterogeneous photodegradation and photo-Fenton degradation; Text S1: Reusability; Text S2: Degradation Scheme of Rh-B.

## References

[1] Al-Tohamy R., Ali S.S., Li F., Okasha K.M., Mahmoud Y.A.G., Elsamahy T., Jiao H., Fu Y., Sun J. (2022). A critical review on the treatment of dye-containing wastewater: Ecotoxicological and health concerns of textile dyes and possible remediation approaches for environmental safety. Ecotoxicol. Environ. Saf..

[2] Iqbal M.A., Akram S., Lal B., Hassan S.U., Ashraf R., Kezembayeva G., Mushtaq M., Chinibayeva N., Hosseini-Bandegharaei A. (2024). Advanced photocatalysis as a viable and sustainable wastewater treatment process: A comprehensive review. Environ. Res..

[3] Nille O.S., Kolekar A.G., Devre P.V., Koparde S.V., Sawat A.H., Sohn D., Patole S.P., Anbhule P.V., Gore A.H., Kolekar G.B. (2025). Nanocarbon eco-hydrogel kit: On-site visual metal ion sensing and dye cleanup, advancing the circular economy in environmental remediation. Analyst.

[4] Wu L., Garg S., Waite T.D. (2024). Progress and challenges in the use of electrochemical oxidation and reduction processes for heavy metals removal and recovery from wastewaters. J. Hazard. Mater..

[5] Chowdhury P.R., Medhi H., Bhattacharyya K.G., Hussain C.M. (2024). Layered double hydroxides derived from waste for highly efficient electrocatalytic water splitting: Challenges and implications towards circular economy driven green energy. Coord. Chem. Rev..

[6] Liu Y., Zhao Y., Wang J. (2021). Fenton/Fenton-like processes with in-situ production of hydrogen peroxide/hydroxyl radical for degradation of emerging contaminants: Advances and prospects. J. Hazard. Mater..

[7] Naghdi S., Shahrestani M.M., Zendehbad M., Djahaniani H., Kazemian H., Eder D. (2023). Recent advances in application of metal-organic frameworks (MOFs) as adsorbent and catalyst in removal of persistent organic pollutants (POPs). J. Hazard. Mater..

[8] Liu H., Tang S., Wang Z., Zhang Q., Yuan D. (2024). Organic cocatalysts improved Fenton and Fenton-like processes for water pollution control: A review. Chemosphere.

[9] Xiao J., Guo S., Wang D., An Q. (2024). Fenton-Like reaction: Recent advances and new trends. Chem.–A Eur. J..

[10] Lu X., Qiu W., Peng J., Xu H., Wang D., Cao Y., Zhang W., Ma J. (2021). A review on additives-assisted ultrasound for organic pollutants degradation. J. Hazard. Mater..

[11] Karim N., Kyawoo T., Jiang C., Ahmed S., Tian W., Li H., Feng Y. (2024). Fenton-like degradation of methylene blue on attapulgite clay composite by loading of iron–oxide: Eco-friendly preparation and its catalytic activity. Materials.

[12] Xu K., Gao Q., Shi S., Liu P., Ni Y., Hao Z., Xu G., Fu Y., Liu F. (2025). Construction of attapulgite-based one-dimensional nanonetwork composites with corrosion resistance for high-efficiency microwave absorption. Int. J. Miner. Metall. Mater..

[13] Wang Q., Liu X., Jiang C., Wang X., Wu L., Li H., Tian W., Feng Y. (2024). Dispersant-assisted rotating liquid film reactor separation strategy for low-grade palygorskite purification with improved dye absorption performance. Clay Miner..

[14] Jiang C., Liu X., Wang X., Wang Q., Li H., Tian W., Ahmed S., Feng Y. (2024). Coupling adsorption and in-situ Fenton-like oxidation by iron-containing low-grade attapulgite clay towards organic pollutant removal: From batch experiment to continuous operation. Green Energy Environ..

[15] Li X., Wang Z., Qin X., Zhang F., Xu C., Tao X., Ren H., Lan X. (2025). Enhancing Fenton-like degradation efficiency over a broad pH range through synergistic interactions among varied acidity sites in M1-O-M2 structures. Sep. Purif. Technol..

[16] Deng J., Zeng Y., Almatrafi E., Liang Y., Wang Z., Wang Z., Song B., Shang Y., Wang W., Zhou C. (2024). Advances of carbon nitride based atomically dispersed catalysts from single-atom to dual-atom in advanced oxidation process applications. Coord. Chem. Rev..

[17] Abid N., Khan A.M., Shujait S., Chaudhary K., Ikram M., Imran M., Haider J., Khan M., Khan Q., Maqbool M. (2022). Synthesis of nanomaterials using various top-down and bottom-up approaches, influencing factors, advantages, and disadvantages: A review. Adv. Colloid Interface Sci..

[18] Nagarajan D., Gangadharan D., Venkatanarasimhan S. (2023). Synthetic strategies toward developing carbon dots via top-down approach. Carbon Dots in Analytical Chemistry.

[19] Etefa H.F., Tessema A.A., Dejene F.B. (2024). Carbon dots for future prospects: Synthesis, characterizations and recent applications: A review. C.

[20] Jassim R.H., Abdalameer N.K., Jebur E.K. (2023). Applications in biomedicine and fabrication using plasma and nanomaterials. Int. J. Nanosci..

[21] Chen B.-R., Roobab U., Madni G.M., Abdi G., Zeng X.-A., Aadil R.M. (2024). A review of emerging applications of ultrasonication in Comparison with non-ionizing technologies for meat decontamination. Ultrason. Sonochem..

[22] Watcharamongkol T., Khaopueak P., Seesuea C., Wechakorn K. (2024). Green hydrothermal synthesis of multifunctional carbon dots from cassava pulps for metal sensing, antioxidant, and mercury detoxification in plants. Carbon Resour. Convers..

[23] Saha N., Gupta S.D. (2025). Innovative textile dye treatment using biomass and metal nanoparticles: An eco-luminescent approach. Biomass Convers. Biorefin..

[24] Lachheb H., Puzenat E., Houas A., Ksibi M., Elaloui E., Guillard C., Herrmann J.-M. (2002). Photocatalytic degradation of various types of dyes (alizarin S, crocein orange G, methyl red, congo red, methylene blue) in water by UV-irradiated titania. Appl. Catal. B Environ..

[25] Kyawoo T., Karim N., Jiang C., Ahmed S., Tian W., Li H., Feng Y. (2024). Facile formation of hierarchical magnesium silicate hydrate microspheres as an adsorbent for the textile dyes. Particuology.

[26] Parveen S., Nazeer S., Chotana G.A., Kanwal A., Batool B., Bukhari N., Yaqoob A., Talib F. (2024). Designing of chitosan/gelatin based nanocomposite films integrated with Vachellia nilotica gum carbon dots for smart food packaging applications. Int. J. Biol. Macromol..

[27] Hu W., Lin S., Cao Y., Feng X., Pan Q. (2022). Preparation and characterization of attapulgite-supported phase change energy storage materials. RSC Adv..

[28] Chen Y., Zhang Y., Zhang J., Zhang J., Feng F., Mu B., Zhang N. (2024). Carbon-dot/attapulgite composite sonocatalytic towards efficient indigo-containing wastewater degradation based on the cavitation mechanism. J. Water Process Eng..

[29] Rasilingwani T.E., Gumbo J.R., Masindi V., Foteinis S. (2024). Removal of Congo red dye from industrial effluents using metal oxide-clay nanocomposites: Insight into adsorption and precipitation mechanisms. Water Resour. Ind..

[30] Zhao Q., Cheng X., Kang J., Kong L., Zhao X., He X., Li J. (2023). Polyvinyl alcohol flame retardant film based on halloysite nanotubes, chitosan and phytic acid with strong mechanical and anti-ultraviolet properties. Int. J. Biol. Macromol..

[31] Azeez S., Shenbagaraman R. (2025). Fourier transform infrared spectroscopy in characterization of bionanocomposites. Characterization Techniques in Bionanocomposites.

[32] Chen X., Chen X., Li B., Ma J., Zhao B., Shao N., Wang Y. (2024). Visible-light-induced degradation of rhodamine B by double Z-scheme magnetic ATP-g-C_3_N_4_/SnFe_2_O_4_/Bi_2_WO_6_ heterojunctions: Performance and mechanism. Surf. Interfaces.

[33] Da Silva Filho P.M., Mariano P.H.R., Andrade A.L., Lopes J.B.A.C., de Azevedo Pinheiro A., de Azevedo M.I.G., de Medeiros S.C., de Vasconcelos M.A., da Cruz Fonseca S.G., Grangeiro T.B. (2023). Antibacterial and antifungal action of CTAB-containing silica nanoparticles against human pathogens. Int. J. Pharm..

[34] Yang X., Wan Y., Zheng Y., He F., Yu Z., Huang J., Wang H., Ok Y.S., Jiang Y., Gao B. (2019). Surface functional groups of carbon-based adsorbents and their roles in the removal of heavy metals from aqueous solutions: A critical review. Chem. Eng. J..

[35] Zhang C., Liu L., Pan Y., Qin R., Wang W., Zhou M., Zhang Y. (2024). Detection methodologies and mechanisms of reactive oxygen species generated in Fenton/Fenton-like processes. Sep. Purif. Technol..

[36] Le T.T., Ahn Y.-Y., Kim K., Chae K.H., Kim S.H., Moon G.-H. (2025). Sustainable mineralization of bisphenol A via iron-oxide-fortified manganese catalysts: Integrating radical and nonradical pathways for advanced wastewater treatment. J. Hazard. Mater..

[37] Huang Y., Yu Q., Li M., Jin S., Fan J., Zhao L., Yao Z. (2021). Surface modification of activated carbon fiber by low-temperature oxygen plasma: Textural property, surface chemistry, and the effect of water vapor adsorption. Chem. Eng. J..

[38] Zhang T., Huang X., Qiao J., Liu Y., Zhang J., Wang Y. (2024). Recent developments in synthesis of attapulgite composite materials for refractory organic wastewater treatment: A review. RSC Adv..

[39] Najdanović S.M., Kostić M.M., Petrović M.M., Velinov N.D., Radović Vučić M.D., Mitrović J.Z., Bojić A.L. (2025). Effect of electrochemical synthesis parameters on the morphology, crystal and chemical structure, and sorption efficiency of basic bismuth nitrates. Molecules.

[40] Li D., Chen Y., Zhou C., Shi C., Xu Z., Miao Z., Xi Z., Han J. (2024). XPS depth profiling of functional materials: Applications of ion beam etching techniques. Mater. Chem. Front..

[41] Chen K., Lv X., Zhang Y., Xin Y., Zhou Z., Chen Y. (2024). Highly efficient removal of uranium (VI) from aqueous solutions by APTES/ATP. Phys. Scr..

[42] Kang X., You Z., Huang Y., Peng J., Zhang J., Ragauskas A.J., Zhang Z., Song X. (2024). Multi-catalytic active site biochar-based catalysts for glucose isomerized to fructose: Experiments and density functional theory study. Adv. Compos. Hybrid Mater..

[43] Li W., Wang H., Sun Z., Wu Q., Xue S. (2021). Si-doped Cu_2_O/SiOx composites for efficient photoelectrochemical water reduction. J. Power Sources.

[44] Jiang W., Chen W., Liao J., Liang X., Xing Y., Wang H., Luo L., Li T., Wang T. (2024). Green synthesis of iron nanoparticles-modified biochar for efficient adsorption of Cd (II) from aqueous solution. Water Air Soil Pollut..

[45] Liang D., Li N., An J., Ma J., Wu Y., Liu H. (2021). Fenton-based technologies as efficient advanced oxidation processes for microcystin-LR degradation. Sci. Total Environ..

[46] Yu H., Liu Y., Xu M., Cong S., Liu M., Zou D. (2021). Hydroxylamine facilitated heterogeneous fenton-like reaction by nano micro-electrolysis material for rhodamine B degradation. J. Clean. Prod..

[47] Chen X.-L., Li F., Chen H., Wang H., Li G. (2020). Fe_2_O_3_/TiO_2_ functionalized biochar as a heterogeneous catalyst for dyes degradation in water under Fenton processes. J. Environ. Chem. Eng..

[48] Najdanović S.M., Petrović M.M., Slipper I.J., Kostić M.M., Prekajski M.D., Mitrović J.Z., Bojić A.L. (2018). A new photocatalyst bismuth oxo citrate: Synthesis, characterization, and photocatalytic performance: Najdanović et al. Water Environ. Res..

[49] Kostić M., Najdanović S., Radović Vučić M., Velinov N., Bojić D., Nikolić G., Bojić A. (2021). A new catalyst with the superior performance for treatment of water polluted by anthraquinone compounds. Bull. Mater. Sci..

[50] Zhang T., Dong L., Du J., Qian C., Wang Y. (2020). CuO and CeO_2_ assisted Fe_2_O_3_/attapulgite catalyst for heterogeneous Fenton-like oxidation of methylene blue. RSC Adv..

[51] Zhang T., Liu Y., Zhang J., Qiao J., Yang X., Li T., Wang Y. (2025). Study on preparation of a pizza-like attapulgite-based composite membrane and its performance on methylene blue and ciprofloxacin removal. Sep. Purif. Technol..

[52] Ai L., Zhang C., Liao F., Wang Y., Li M., Meng L., Jiang J. (2011). Removal of methylene blue from aqueous solution with magnetite loaded multi-wall carbon nanotube: Kinetic, isotherm and mechanism analysis. J. Hazard. Mater..

