# Supplementary material for: Carbon-Modified Attapulgite Composite for Rapid Rhodamine B Degradation: High Adsorption Capacity and Photo-Fenton Efficiency
Source: Materials (Basel). 2026 Jan 30;19(3):554. doi: 10.3390/ma19030554 (PMC12898179; doi:10.3390/ma19030554)
Supplement: Supplementary file 1 [file materials-19-00554-s001.zip › materials-3997641-supplementary.pdf]

materials

MDPI

Supporting Information

Carbon-Modified Attapulgite Composite for Rapid Rhodamine B Degradation: High Adsorption Capacity and Photo-Fenton Efficiency

Naveed Karim [1], Tin Kyawoo [1], Saeed Ahmed [2], Weiliang Tian [3], Huiyu Li [1] and Yongjun Feng [1,3,*]

[1] State Key Laboratory of Chemical Resource Engineering, Beijing University of Chemical Technology, Beijing 100029, China; karim@buct.edu.cn (N.K.); huiyuli@mail.buct.edu.cn (H.L.)
[2] Department of Biological & Chemical Sciences, University of Rasul, Mandi Bahauddin, 50370, Pakistan
[3] College of Chemistry and Chemical Engineering, Tarim University, Alar, 843300, PR China
* Correspondence: yjfeng@mail.buct.edu.cn; Tel.: +86-10-6443-6992

Table S1. Binding energy peak percentage of Fe^{2+} and Fe^{3+} in A-ATP@C composite.

	**Binding Energy (Fe^{2+})**	**(%)**	**Binding Energy (Fe^{3+})**	**(%)**
Before Degradation	712.5	38.05	722.2	15.62
	715.1	19.07	728.2	5.63
	724.9	21.63		
After Degradation	712.7	37.40	722.2	17.43
	715.4	17.54	728.2	7.14
	724.5	20.29		

Table S2. Comparison study for Heterogeneous photodegradation and photo-Fenton degradation.

**Sample**	**Heterogeneous Fenton (efficiency/time)**	**Photo-Fenton Degradation (efficiency/time)**
A-ATP	61.3% / 20–100 min	83.5% / 20–50 min
A-ATP@CTAB	70.6% / 20–100 min	94.6% / 20–50 min
C-A-ATP@CTAB	76.2% / 12–20 min	100% / 12–20 min

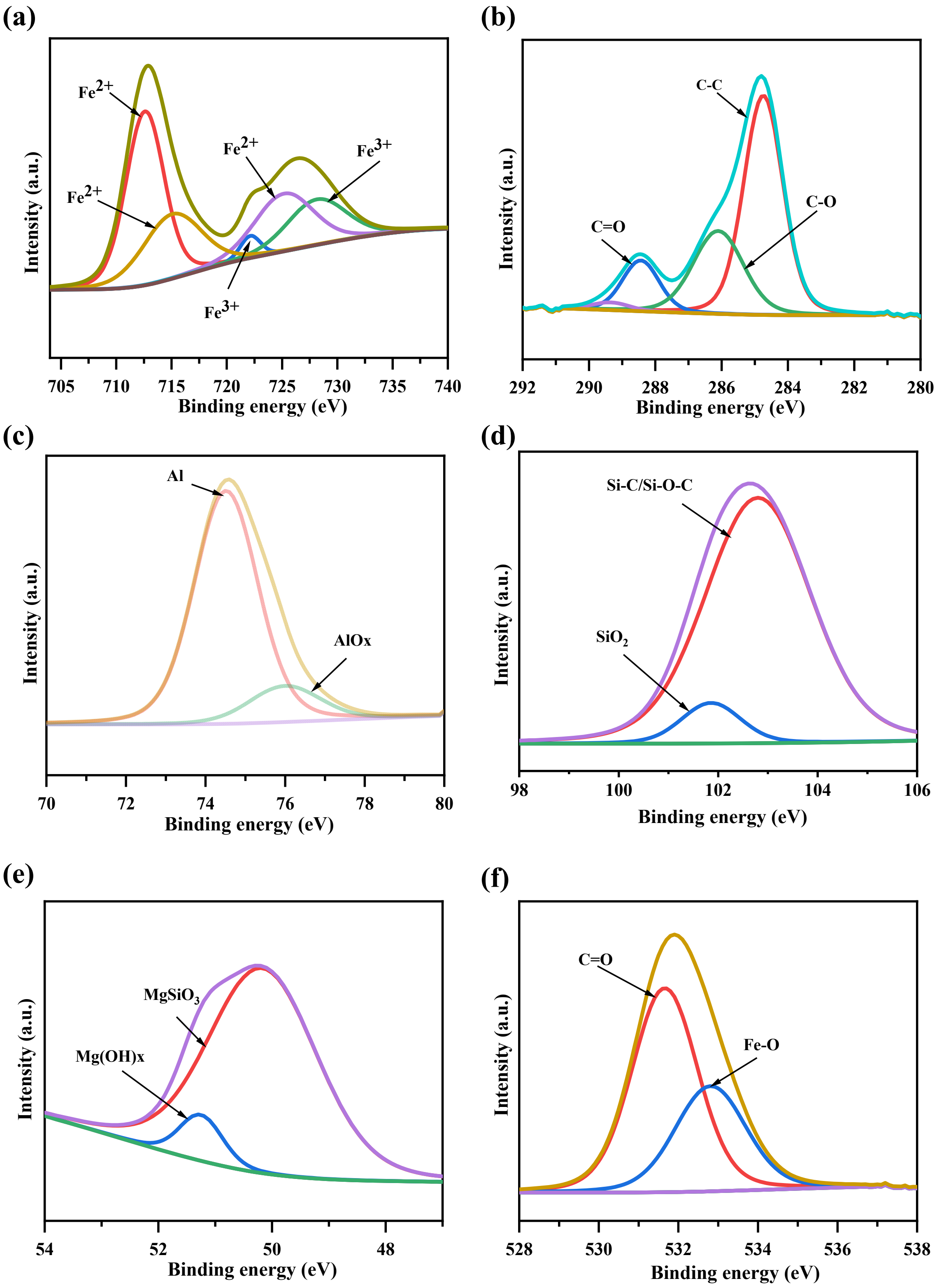

Figure S1. After degradation: (**a**) types of iron; (**b**) oxygen functionalities; (**c**) aluminum and AlO_x; (**d**) nature of silicon functional groups; (**e**) nature of magnesium functionalities; (**f**) carbon functionalities in C-A-ATP@CTAB composite.

Text S1. Reusability

Reusability and constancy are critical factors for catalysts in industrial applications. This study evaluates the reusability of the C-A-ATP@CTAB nanocomposite in consecutive Fenton and Photo Fenton reactions for the degradation of Rh-B. Following each response

run, the degradation efficiency was measured, and the results indicated a modest decline in efficiency throughout the subsequent runs. The degradation efficacy was reduced from 99.8% to 88% after the C-A-ATP@CTAB nanocomposites were recycled four times. This suggests that the repurposed nanocomposites maintained a respectable level of catalytic activity and showed high stability for the degradation of organic contaminants. Following four cycles of use, several characterization tests were carried out to further verify the catalyst's stability.

The recycling efficiency of C-A-ATP@CTAB was assessed after each degradation cycle. The composites were recovered using ethanol rinsing until neutralization, followed by drying at 70 °C for 12 h. By the fifth cycle, a slight reduction of 5–10% in maximum degradation capacity was observed in Figure S2d. However, as shown in Figure S2b, the functional groups remained ~100% intact after recycling, indicating that the degraded dye was removed entirely following the Fenton degradation process. This conclusion was further supported by the FTIR spectra, where no significant changes in intensity were detected before and after degradation for each adsorbate sample. A relative decrease in the full-width at half maximum (FWHM) of the XRD spectrum for A-ATP, A-ATP@CTAB, and C-A-ATP@CTAB after the Fenton and photo-Fenton degradation suggests a reduction in silicate concentration. At the same time the lattice structure remained stable Figure S2c.

Furthermore, the FTIR, XRD, and XPS analyses demonstrated that the structural and chemical composition of the composite remained unchanged even after five degradation cycles, confirming its stability and reusability. Additionally, the atomic composition of iron oxide remained consistent after degradation, verifying the chemical stability of C-A-ATP@CTAB during the Fenton process. The increased atomic fraction of adsorbates suggests that Rh-B adsorption/degradation occurred through chemical interactions, a conclusion further reinforced by the synergistic relationship between adsorption/degradation behavior and XRD results. Moreover, the findings indicate that C-A-ATP@CTAB composites outperform R-ATP in terms regarding dye adsorption/degradation and degradation efficiency, which can be attributed to their higher surface area and mixed porous structure, facilitating more effective pollutant removal.

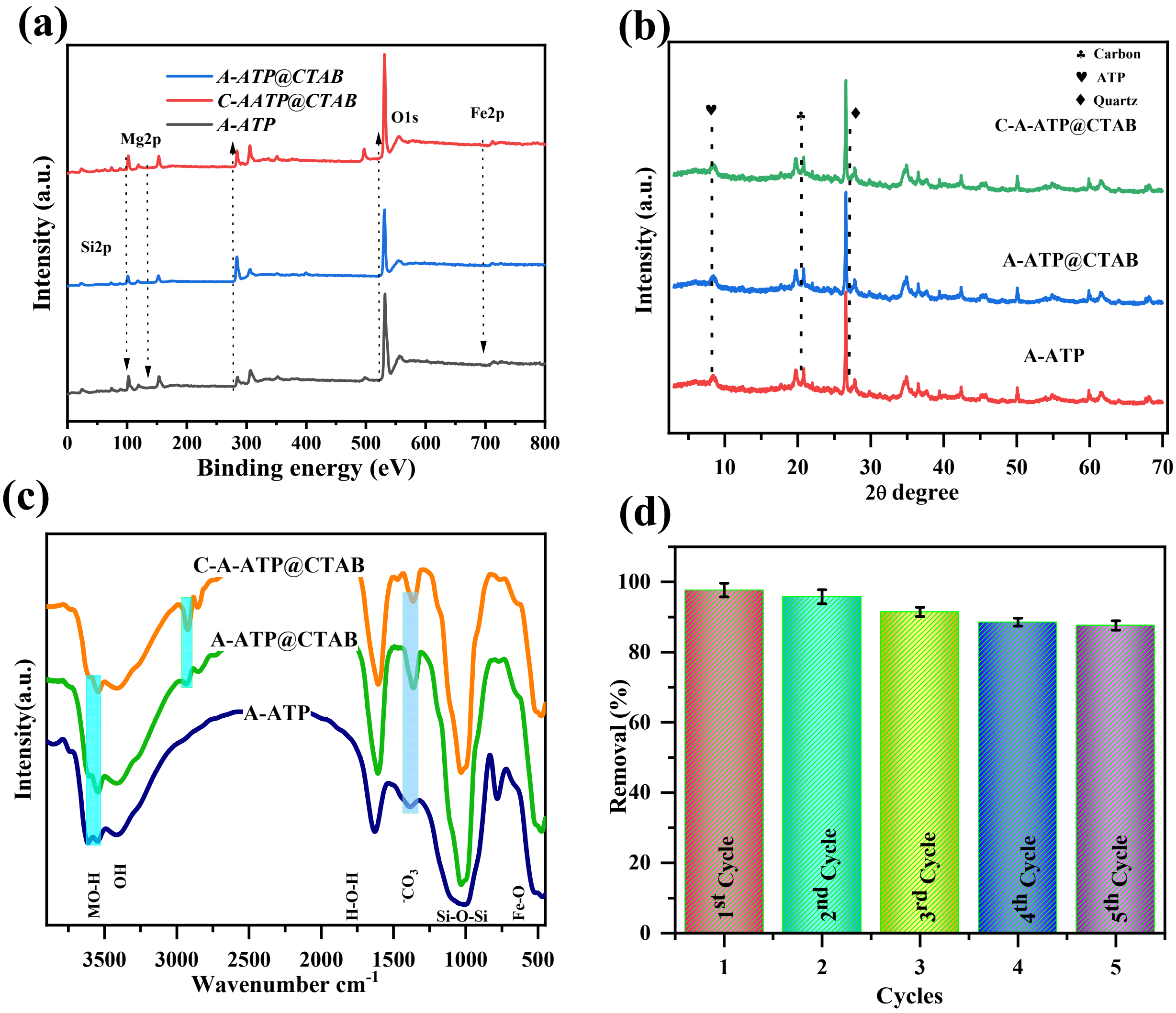

Figure S2. Reusability of CDs-A-ATP@CTAB nanocomposite after washing with ethanol and neutralization (**a**–**d**), XPS, XRD, FTIR, and reused cycle efficacy.

Text S2. Degradation Scheme of Rh-B

C-A-ATP@CTAB composite was determined to eliminate the stubborn (Rh-B) dye-polluting wastewater in Figure S3. As the experiment commenced, the composite C-A-ATP@CTAB, generated reactive oxygen species (ROS) that swiftly attacked Rh-B molecules (m/z = 443). The first transformation began through N-demethylation, stripping away ethyl groups and forming intermediates: Product 1 (m/z = 415), Product 2 (m/z = 415), Product 5 (m/z = 387), Product 6 (m/z = 387), and Product 9 (m/z = 331); progressively breaking down the dye's structure.

Meanwhile, pathway II introduced hydroxylation, forming Product 3 (m/z = 459), which further degraded into Product 7 (m/z = 379) and eventually Product 9 through dihydroxylation. In Pathway III, the dye’s conjugated structure shattered, leading to Product 4 (m/z = 339), which fragmented into Product 8 (m/z = 299) and Product 10 (m/z = 271) through further hydroxylation and demethylation. As the molecular breakdown continued, the smaller intermediates—Product 11 (m/z = 274), Product 12 (m/z = 196), Product 13 (m/z = 258), Product 14 (m/z = 244), Product 15 (m/z = 230), Product 16 (m/z = 315), and Product 17 (m/z = 106)—formed through C–N bond cleavage, setting the stage for complete mineralization. These fragments underwent ring-opening reactions, leading to final products such as Product 18 (2-hydroxypentanedioic acid, m/z = 148), Product 19 (n-nonane, m/z = 128), Product 20 (benzoic acid, m/z = 122), Product 21 (butan-1,3-diol, m/z = 90),

and Product 22 (3-amino-1,3-butadien-1-ol, m/z = 85). Eventually, all intermediates were mineralized through continuous oxidation into CO_2 and H_2O, leaving behind only purified water shown in Figure S3. As the researcher observed the results, they noted that after multiple cycles, C-A-ATP@CTAB retained its catalytic efficiency, proving its robustness and reusability.

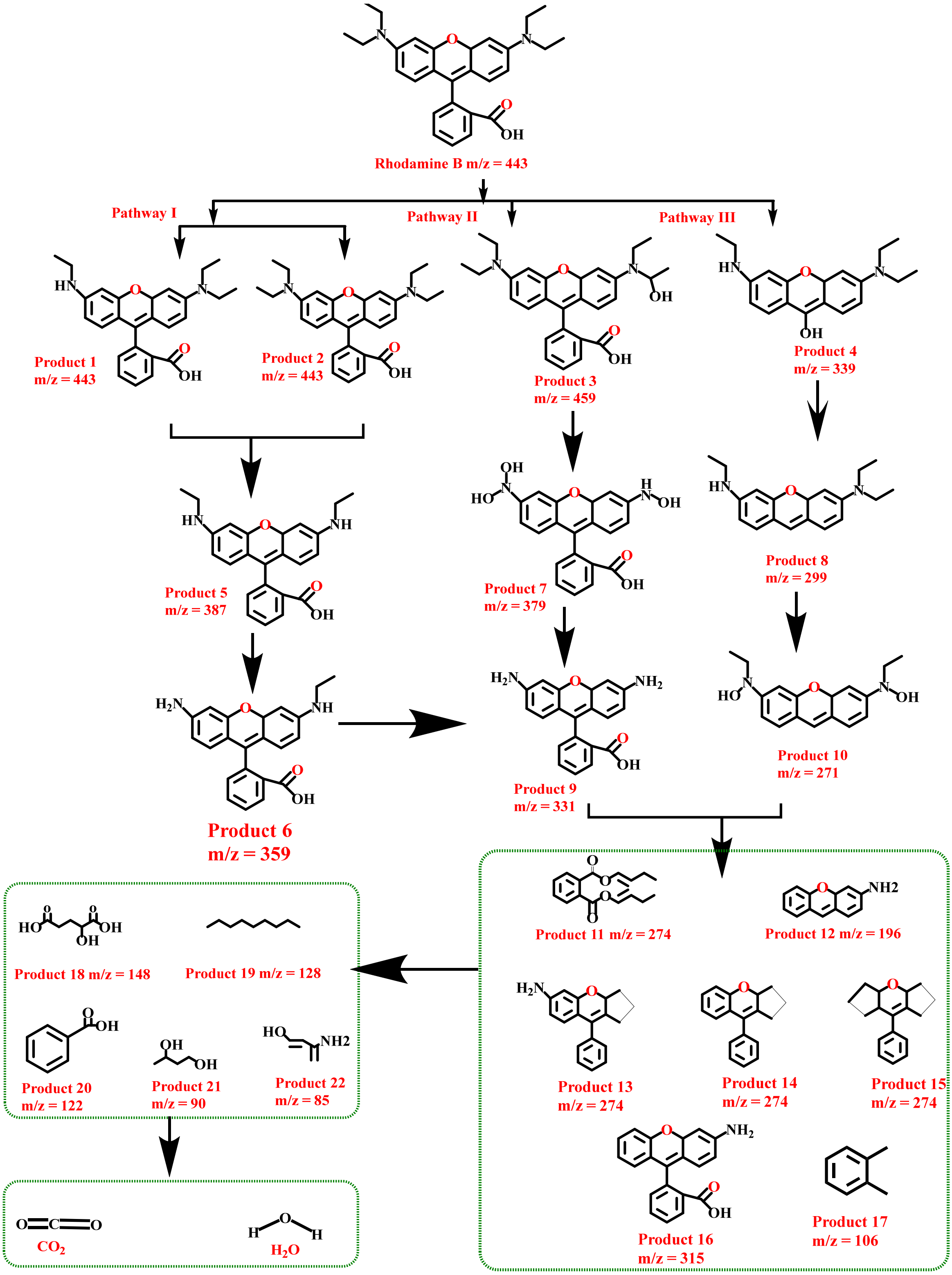

Figure S3. LC-MS possible degradation pathways of Rh-B in the C-A-ATP@CTAB.
